# Supplementary material for: Characterization and isotherm data for adsorption of Cd2+ from aqueous solution by adsorbent from mixture of bagasse-bentonite
Source: Data Brief. 2017 Nov 21;16:354–60. doi: 10.1016/j.dib.2017.11.060 (PMC5723263; doi:10.1016/j.dib.2017.11.060)
Supplement: Supplementary file 1 — Supplementary material [file mmc1.docx]

**Conflict of Interest Form**

There is no conflict of interest on this research work.
